# Ultrasound-Assisted Processing of Amorphous Hydrated Carbon Oxide from Plant Origin: Properties and Biocompatibility of Conductive Nanocomposites as a Proposal for Food Packaging

**DOI:** 10.1021/acsomega.5c13160

**Published:** 2026-03-17

**Authors:** Kelvi W. E. Miranda, Francislei S. A. Santos, Francisco Carlos C. S. Salomão, Gabrielle A. Freire, Maria Kueirislene A. Ferreira, Jane E. S. A. Menezes, Antonio G. Souza Filho, Melvin Pascall, Maria do Socorro R. Bastos, Lucicléia B. Vasconcelos

**Affiliations:** † Laboratory for Research and Innovation in Plant Products and Packaging, Department of Food Engineering, Federal University of Ceará, Fortaleza, Ceará 60356-000, Brazil; ‡ Department of Industrial Processes and Chemical Engineering, Federal Institute of Bahia, Campus Salvador, Salvador, Bahia 40110-150, Brazil; § Science and Technology Center, State University of Ceará, Fortaleza, Ceará 62930-000, Brazil; ∥ Department of Physics, Federal University of Ceará, Fortaleza, Ceará 60455-900, Brazil; ⊥ Department of Food Science and Technology, 2647 The Ohio State University, Columbus, Ohio 43210, United States; # Packaging Laboratory, EMBRAPA Tropical Agroindustry, Fortaleza, Ceará 60511-110, Brazil

## Abstract

The sustainable production of conductive carbon nanomaterials for food-packaging applications remains limited by dispersion instability and safety concerns. Herein, we investigate the ultrasound-assisted processing of plant-derived hydrated amorphous carbon oxide (HACO) to obtain stable, conductive, and biocompatible nanostructured dispersions. Ultrasonic cavitation was applied in a hydroalcoholic medium, and a full 2^3^ factorial design was used to evaluate the effects of sonication amplitude and time on particle size, polydispersity index (PDI), and zeta potential, together with acoustic parameters. Optimized conditions produced dispersions with particle sizes below 150 nm and PDI <0.30 after 24 h, with 1% (v/v) ethanol acting as an effective stabilizer. Raman spectroscopy confirmed an amorphous carbon structure (*I*_D_/*I*_G_ = 0.87), while atomic force microscopy (AFM) and FEG-SEM revealed nanostructured aggregates. Cyclic voltammetry showed peak currents above 30 μA and Δ*E*_p_ values between 70.8 and 95.2 mV, indicating good electrical conductivity. Although no acute toxicity or mortality was observed in adult zebrafish after 96 h of exposure, the highest concentrations of HSACO (3.0%, v/v) and the pure material induced sublethal behavioral changes. The novelty of this work lies in the combination of renewable, plant-based amorphous carbon oxide and controlled ultrasonic cavitation to produce safe and conductive nanostructures, highlighting HACO as a promising sustainable filler for smart food-packaging nanocomposites.

## Graphical abstract

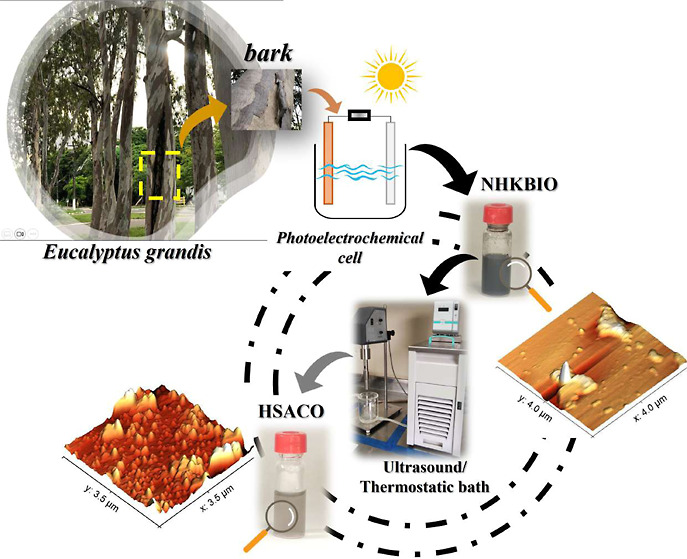

## 1 Introduction

Nanotechnology encompasses the technological use of nanomaterials and systems assembled and studied by nanoscience. It also develops highly organized nanostructures for new materials and/or improvements in their properties and characteristics.^1^ With this, research has been intensified in the search for sustainable, ecofriendly materials (in the production route and finished product) that are biocompatible with other materials.^2–4^

Carbon-based nanomaterials, such as graphene and similar 2D materials, are widely used and studied in polymeric matrices as nanocomposites and considered nanomaterials of great versatility and applicability due to their complexity and a set of improved mechanical, optical, thermal, magnetic, and/or electrical properties.^3,5–7^ In addition, their biocompatibility under certain circumstances and good chemical stability make them promising for many applications.^2^ This combination of properties makes carbon-based material an emerging technological innovation with applications in various areas of knowledge: biomedicine,^8^ construction,^9^ agribusiness,^10^ cosmetics,^11^ pharmacology,^12^ and functional food materials.^13^ Graphene, in particular, is noted for its exceptional electrical, thermal, and mechanical properties, associated with its highly ordered two-dimensional structure.^14,15^

With technological advances in the field of nanotechnology, there is an excellent opportunity to obtain materials such as graphene with a good sustainable profile (no generation of processing waste), low cost, and high quality.^2,16^ The traditional chemical and physical routes for obtaining graphene involve (i) mechanical peeling of graphite (adhesive tape) and/or anodic bonding;^17,18^ (ii) thermal deposition; (iii) chemical and thermal reduction of graphene oxide;^19,20^ (iv) chemical exfoliation from graphite (Hummers method);^21^ and (v) chemical vapor deposition (CVD), which uses metallic substrates of gaseous hydrocarbon precursors.^22^

In this context, these methods are economically unfeasible on an industrial scale, as they use expensive resources and conditions such as high temperatures, a large number of synthesis steps, and/or the use of high-cost reaction precursors.^16^ Therefore, searching for low-cost commercial methods and/or materials that allow standardization and scalable production is necessary and has a positive economic and environmental impact.^23^ Thus, liquid-phase exfoliation (LPE) is the most popular and simple processing and synthesis method, widely used in obtaining graphene from graphite on a large scale.^24^ However, the choice of critical parameters such as sonication mode, time, and power directly influences the quality and characteristics of the cavitated carbonaceous material.^25^

This research introduces the innovation of NHKBIO, a carbon-based nanocomposite comprising hydrated amorphous carbon and plant-derived graphene oxide (GO) synthesized via the NHK (nano-hope-key) route. This process is based on the photoelectrochemical redox of *Eucalyptus grandis* bark biomass under ambient temperature and pressure conditions powered by solar energy, representing a clean technology alternative to traditional methods such as chemical vapor deposition (CVD).^26^ While conventional routes require high temperatures and aggressive reagents and result in significant environmental impacts and operational costs, the NHK route mitigates mining liabilities and greenhouse gas emissions,^26,27^ enabling the sustainable and scalable production of carbon nanostructures. It should be noted that specific technical details are not further detailed herein, as they are protected under patent application n^o^ BR102024023159-7.

Despite advances in obtaining carbon nanomaterials, the search for sustainable, scalable, and economical methods from renewable sources remains challenging. In this context, hydrated amorphous carbon oxide (HACO) from the eucalyptus plant has emerged as a promising material. This study aimed to investigate the processing of HACO via ultrasonic cavitation in a hydroalcoholic medium, optimizing the conditions for obtaining stable nanostructured dispersions and characterizing their functional properties and biological safety. To this end, a full factorial design (FFD) was used to statistically evaluate the influence of the sonication amplitude and time on the dispersion stability (particle size and PDI). The optimized material was subjected to structural (Raman), morphological (atomic force microscopy (AFM) and SEM), and electrochemical (cyclic voltammetry) characterization. In addition, acute toxicity was assessed by using an in vivo zebrafish (*Danio rerio*) model. This work aimed to provide fundamental insights into the processing and properties of HACO, proposing it as an emerging, viable, and safe material for developing conductive nanocomposites with particular interest in food packaging applications.

## 2 Experimental Methods and Materials

### 2.1 Carbonaceous Material

Hydrated amorphous carbon oxide (HACO), commercially known as NHKBIO, was kindly supplied by AdpCLEAN Ltd. (Bahia, Brazil). This material is synthesized using the “U” photoelectrochemical route, based on the NHK (nano-hope-key) process, from *Eucalyptus grandis* extract, under normal temperature and pressure (NTP). NHKBIO has a density of between 1.25 and 1.35 g cm^–3^ and a heterogeneous composition.

The “U” photoelectrochemical route used in the NHK process consists of a synthesis method under mild environmental conditions, which combines light excitation and controlled electric current to promote the transformation of *Eucalyptus grandis* extract into HACO. The developed product is the subject of patent application n. BR102024023159-7, which is still under review. Technical details are described in the corresponding patent application, held by AdpCLEAN Ltda. (Brazil).

### 2.2 Materials

Absolute ethyl alcohol P.A. 99.8% pure (*M*_w_ = 46.07 g/mol, CAS 64-17-5, density 0.7890–0.7915 g/cm^3^) was from Exodus Scientific, Brazil. Ultrapure water was obtained (Milli-Q). Zebrafish (*Danio rerio*) 90–120 days old (age), 3.5 ± 0.5 cm (length), and 0.4 ± 0.1 g (weight), both sexes and wild-caught, acquired in Fortaleza, CE, were kept in glass aquariums at 25 ± 2 °C on 24 h light–dark cycles.

### 2.3 Behavior of HACO Suspension in Hydroalcoholic Medium

The samples were prepared with 18.16 μL of NHKBIO suspension, 0.4 mg/mL, added to 50 mL of ultrapure water (Milli-Q), 0.4 mg/mL of NHKBIO and a stabilizing agent for the carbon oxide suspension, and absolute ethyl alcohol in different concentrations: 1.0, 3.0, 5.0, and 7.0% (v/v), according to a methodology adapted from Wang et al.^28^ In this case, the ethanol after ultrasonic cavitation will promote the reduction of the surface tension of the cavitated material, maintaining the suspension’s stability.^29^

The suspensions were subjected to low-frequency ultrasonic cavitation (24 kHz) in an ultrasonic cleaner (model UP400S, Hielscher, GER) coupled to a circulating thermostatic bath (model F-25, Julabo, GER) at 15 °C to maintain the internal temperature of the hydrated amorphous carbon oxide hydroalcoholic suspension (HSACO) at 24 ± 2 °C. A 22 mm diameter probe was standardized at a height of 15 mm (in relation to the base of the jacketed beaker) in a liquid medium (Figure 1). The behavior of the stabilizing agent in the suspension was analyzed over 120 h using dynamic light scattering (DLS) and polydispersity index (PDI) analysis on the Zetasizer Nano ZS equipment (Malvern Panalytical, Malvern, UK).

**Figure 1:**
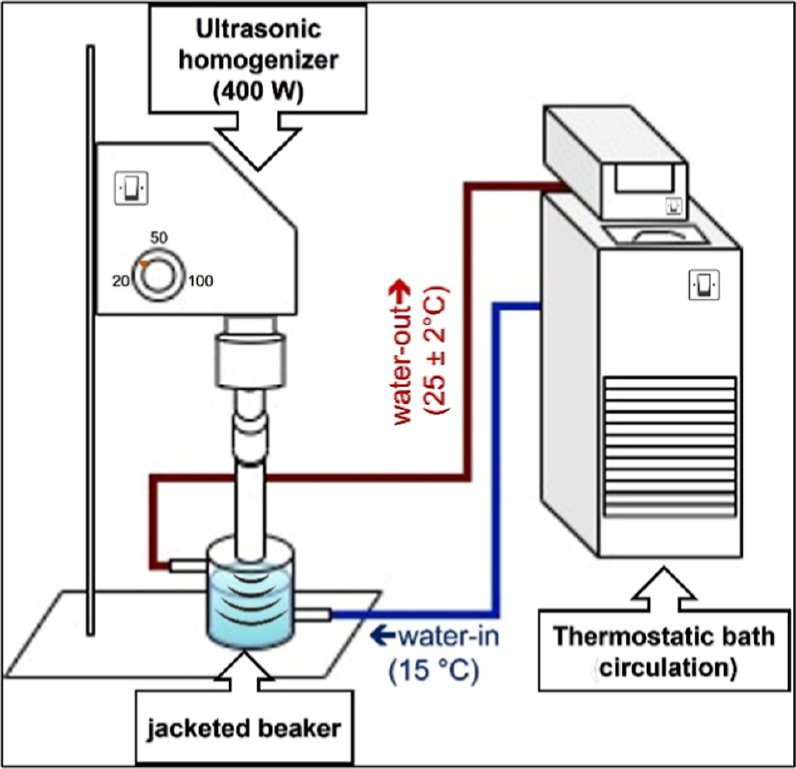
Schematic of the ultrasonic cavitation system with tip ultrasound coupled to the circulating thermostatic bath.

### 2.4 Factorial Design in Ultrasound Processing

The nanometric particles of HSACO at 1% (v/v) ethanol were obtained using a full factorial design of 2^3^. The influence of the main parameters of the sonication optimization process (amplitude and time) was studied using 11 randomized experimental combinations (Table 1 and Supporting Information, Table S1).

**Table 1:** 2^3^ Full Factorial Design for Evaluating the Main Parameters of Sonication on HSACO

factors	unit	level		
		–1	0	1
amplitude	%W	30	50	70
time	min	60	120	180

The effects and interactions of the parameters were studied by using Pareto diagrams and contour surface plots, respectively. To determine the hydrodynamic size distribution of the particles in the HSACO, dynamic light scattering (DLS), polydispersity index (PI), and zeta potential (ζ) in Zetasizer Nano ZS equipment (Malvern Panalytical, Malvern, UK) under conditions after 24 h in standby (P24) were determined.

Ultrasonic cavitation was carried out as described in Section 2.3 (Figure 1). The samples were prepared in 50 mL of ultrapure water (Milli-Q) with 18.16 μL of NHKBIO. The experiments were carried out in duplicates and characterized in terms of acoustic power, specific energy, and ultrasound intensity,^30^ using eqs 1–3, respectively.

1 $$acoustic\;power\left(W\right)=mC_{p}\left(\frac{dT}{dt}\right)$$

2 $$specific\;energy\left(\frac{kJ}{g}\right)=\frac{acoustic\;power\times processing\;time}{mass}$$

3 $$ultrasound\;intensity\left(\frac{W}{{cm}^{2}}\right)=\frac{4\times acoustic\;power}{\pi D^{2}}$$

where mass corresponds to the mass of the sample (g); *C*_p_, specific heat (J/g.°C); $\frac{dT}{dt}$ , the temperature rate (°C/s); and *D*, the diameter of the probe (cm). Table S2 in the Supporting Information shows the characteristics of the ultrasonic process through acoustic power, specific energy, and ultrasonic intensity determined for each nominal power applied in the treatments.

### 2.5 Characterization of HSACO-8

Morphological and topographical evaluation of the HSACOs was determined by atomic force microscopy (AFM) on Asylum MFP-3D BIO equipment (Oxford Instruments, CA, USA), in air and in intermittent contact mode, using NCHR-W tips (Nano World Innovative Technologies) with a typical AFM tip radius of curvature of less than 8 nm. Approximately 5 μL of the HSACO-8 sample was placed on the Si substrate and exposed to red light for 1–2 min to ensure complete evaporation of the solvent (water/ethanol). The results were analyzed using Gwyddion 2.65, the software in the instrument.

For the Raman spectroscopy analysis, we used an Alpha 300 system from Oxford Instruments, equipped with an ultrahigh-performance spectrometer (UHTS) with a final spectral resolution of less than 1 cm^–1^. The Raman response was obtained using the 532 nm line of an Nd/YAG laser, focused on the sample with a 10× magnification objective, a working distance of 6.7 mm, and a numerical aperture of 0.25. The laser power on the sample surface was kept below 0.6 mW, and the accumulation time was 2 s. The sample prepared for AFM was used for Raman spectroscopy, maintaining the homogeneity of the readings, for the purpose of spectral mapping and analysis.

The micrographs were taken using a Quanta FEG microscope using an electron acceleration voltage of 20 kV. The sample, approximately 32 μL, was placed in drop-shaped aluminum supports and subjected to red light for 5 min to ensure complete evaporation of the solvent from the suspension (water/ethanol). Silver coating was not necessary due to the conductive nature of the material (behavior observed in preliminary tests not presented).

The electrochemical properties of the samples were studied using an Autolab PGSTAT 12 potentiostat/galvanostat (Ecochemie, Netherlands) with Nova 2.1 software (Metrohm).^31,32^ A Faraday cage and an electrochemical cell with three electrodes were used for the measurements: a glassy carbon disk electrode (GCE) as the working electrode (*A* = 0.07069 cm^2^ for Ø = 3.0 mm), a helical platinum auxiliary electrode, and Ag/AgCl | KCl 3 mol/L as the reference electrode. The current density (j) was obtained from the cyclic voltammetry (CV) data by normalizing the measured current (I) by the surface area of the working electrode. An aqueous electrolyte medium of potassium ferricyanide (K_3_[Fe­(CN)_6_]) 4.0 mmol/L and KCl 1.0 mol/L in a potential window of −0.30 to 0.80 V at 50 mV/s was also used. The GCE was prepared and cleaned according to Gonzaga et al.^32^ with adaptations. The samples were placed on the GCE electrode by using the drop-casting technique.

### 2.6 Acute Toxicity and Novel Tank Test

The studies were carried out according to an analysis protocol described by Carneiro Romão et al.^33^ Acute toxicological analysis (96 h) was carried out with a total of 36 fish divided into 6 groups. These were treated orally with 20 μL of hydroalcoholic carbon solution at concentrations of 0.75%, 1.50%, and 3.00% (v/v), NHKBIO (pure carbon, commercial), and controls (Milli-Q water and 3.0% hydroalcoholic solution, v/v). The fish were tested for analysis of their mortality rates over a 96 h period. At 24 h intervals, the numbers of fishes killed in each group were recorded.^34^ The LC_50_ (lethal concentration capable of killing 50% of the fish) was determined using the probit mathematical method with a 95% confidence interval. The fish were normally fed twice a day with spirulina (flakes). The study was approved by the Animal Use Ethics Committee of the State University of Ceará (CEUA-UECE; *n*° 04983945/2021), in accordance with the Ethical Principles of Animal Experimentation.

After 60 min of oral ingestion of the treatments, the fishes were subjected to the novel tank test to study locomotion and anxiety-like behavior (novel tank).^35,36^ The fish were transferred to individual tanks, divided into three equal horizontal areas (lower, middle, and upper) 4 cm high, and recorded on video (5 min). Behavior was recorded using an 8.0 MP camera (Samsung), and the videos were analyzed using the ZebTrack-UFRN software (version 2021).^35^ The following parameters were considered: total distance traveled, average speed in motion, total immobility time, time and distance traveled in the upper area, and latency to enter the upper area.^36^ All analyses were carried out using GraphPad Prism version 8.0 software, with a statistical significance level of 5%.

### 2.7 Statistical Analysis

The data were statistically analyzed using Statistica Software Version 12 (StatSoft, Inc., USA, 2011). The data were analyzed using one-way analysis of variance (ANOVA), followed by Tukey’s method at a 5% significance level.

## 3 Results and Discussion

By using surfactant solutions (ionic or nonionic) in suspensions, it can ensure that the suspended materials do not agglomerate.^28^ Figure 2 shows the influence of ethyl alcohol on the particle size and polydispersity index characteristics of the cavitated carbonaceous material.

**Figure 2:**
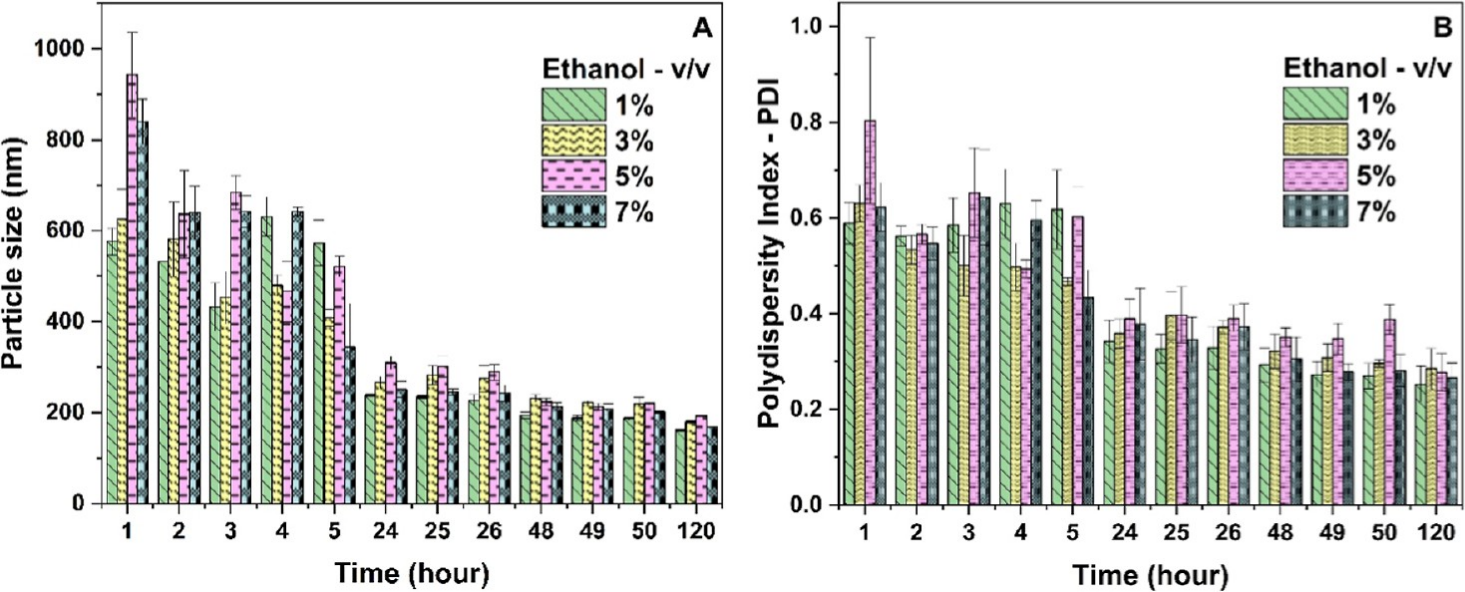
Behavior of HSACO over 120 h in relation to different concentrations of the dispersing agent, ethanol, for (A) particle size and (B) polydispersity index (PDI) (*n* = 3).

Over time, the particle size and PDI decreased, as shown in Figure 2. However, from 24 h onward, after the ultrasonic cavitation process, no statistical differences were observed in particle size in the suspensions (*P* > 0.05). However, the PDI of the samples varied (*P* < 0.05), with only 1.0% having a PDI <0.30. Also, there was no significant difference between the 1.0% and 3.0% treatments (*P* >0.05). The literature reports that a PDI ≤0.30 of particle suspensions shows a homogeneous distribution of sizes.^37^ Although the results indicate stability of the dispersed system after 24 h of ultrasonic processing, we recognize that for applications in packaging matrices, long-term stability is essential to ensure adequate performance and safety. Therefore, further tests with extended periods are necessary to validate the material’s resistance to sedimentation, aggregation, and physicochemical changes during prolonged storage.

Varying the water–ethanol ratio may have made it easier to maintain the dispersion of the HSACO nanostructures. According to Martis et al.,^29^ ethanol helps reduce the surface tension of the cavitated material. Furthermore, the organic solvent shows compatibility for process steps in film formation using the casting method. Literature shows that incorporating ethanol into water improves dispersion stability, increasing exfoliation efficiency by overcoming van der Waals forces and regulating cavitation and solvation energy when applied during the ultrasonication process.^38,39^

Therefore, conditions lower than 7.0% showed a better particle size distribution and PDI performance. The non-agglomeration of the carbon oxides was attributed to the osmotic repulsion force being greater than the van der Waals forces.^40^ Wang et al.^28^ observed similar behavior when using ethanol in solutions with nonionic or ionic surfactants and graphene. These authors reported an increase in the stability and yield of the exfoliation process. This occurred because of the interaction between surfactant–graphene–surfactant, where the solvent (ethanol) filled the voids in the material, thus reducing the enthalpy of the mixture.^28^

### 3.1 Characterization of HSACO

Reducing particle size can enhance material characteristics such as physicochemical properties, optics, viscosity, sedimentation speed, and bioactivities.^41^ The ultrasonic cavitation mechanism using tip ultrasound (Figure 1) provides stable material dispersion when associated with stabilizing agents such as organic solvents and ethanol.^3,28^ In addition, the shear stress applied, combined with time and sonic power, can promote changes to the structural characteristics of the material,^3^ as shown in the response surfaces in Figure 3.

**Figure 3:**
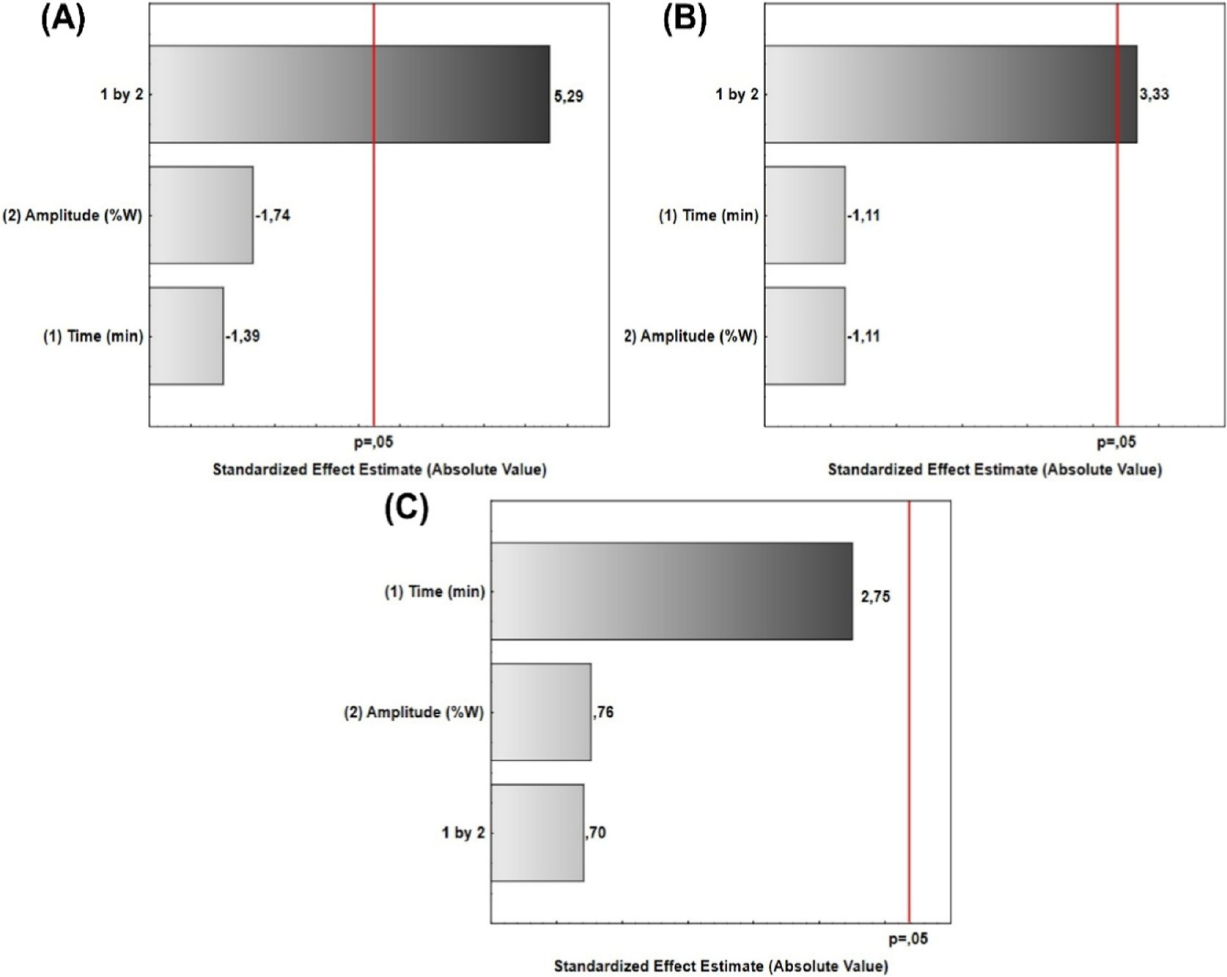
Standardized effects of time and amplitude factors in ultrasonic cavitation on (A) particle size, (B) polydispersity index (PDI), and (C) zeta potential (ζ).

Figure 3 shows the standardized effects in the Pareto chart for the ultrasonic cavitation process under particle size, PDI, and ζ. Therefore, time and amplitude combined are the most significant factors influencing the particle size and PDI of HSACO (*P* < 0.05). In contrast, ζ was not affected by the individual or combined factors. In this case, the physical behavior of the particles depended on the electrical state of the material’s interphases; the larger the surface area, the greater the charge, and the better the relationship with the hydroalcoholic solution.^42^ Similar behavior was found in the literature when graphene dispersions in organic and aqueous media were analyzed.^43^ Table 2 shows the particle size obtained through the experimental design (Supporting Information, Table S1).

**Table 2:** Particle Size Obtained through HSACO Acoustic Cavitation

treatment	amplitude (%W)	time (min)	particle size (nm)
1	30	60	294.0 ± 19.5^a^
2	70	180	239.1 ± 3.4^b^
3∗^a^	50	120	198.7 ± 4.2^ce^
4	30	180	176.8 ± 2.0^df^
5	70	60	170.6 ± 3.2^f^
6∗^a^	50	120	200.1 ± 2.0^ce^
7	50	180	170.9 ± 3.1^f^
8	30	120	183.9 ± 0.9^def^
9	70	120	187.8 ± 4.5^def^
10	50	60	191.1 ± 4.9^cde^
11∗^a^	50	120	208.1 ± 1.8^c^

a (∗) Corresponds to the central component of the experimental design.

Table 2 shows the dispersibility of HSACO after 24 h of rest following the sonication process. The data show stability in the supernatant fraction. However, sedimentation of aggregate material, including semi- and microstructured components, was observed. This behavior suggests that the stability of the dispersion is associated with the maintenance in suspension of the nanostructured fraction, whose characteristics are influenced by the ultrasonic cavitation parameters (Figure 4).

**Figure 4:**
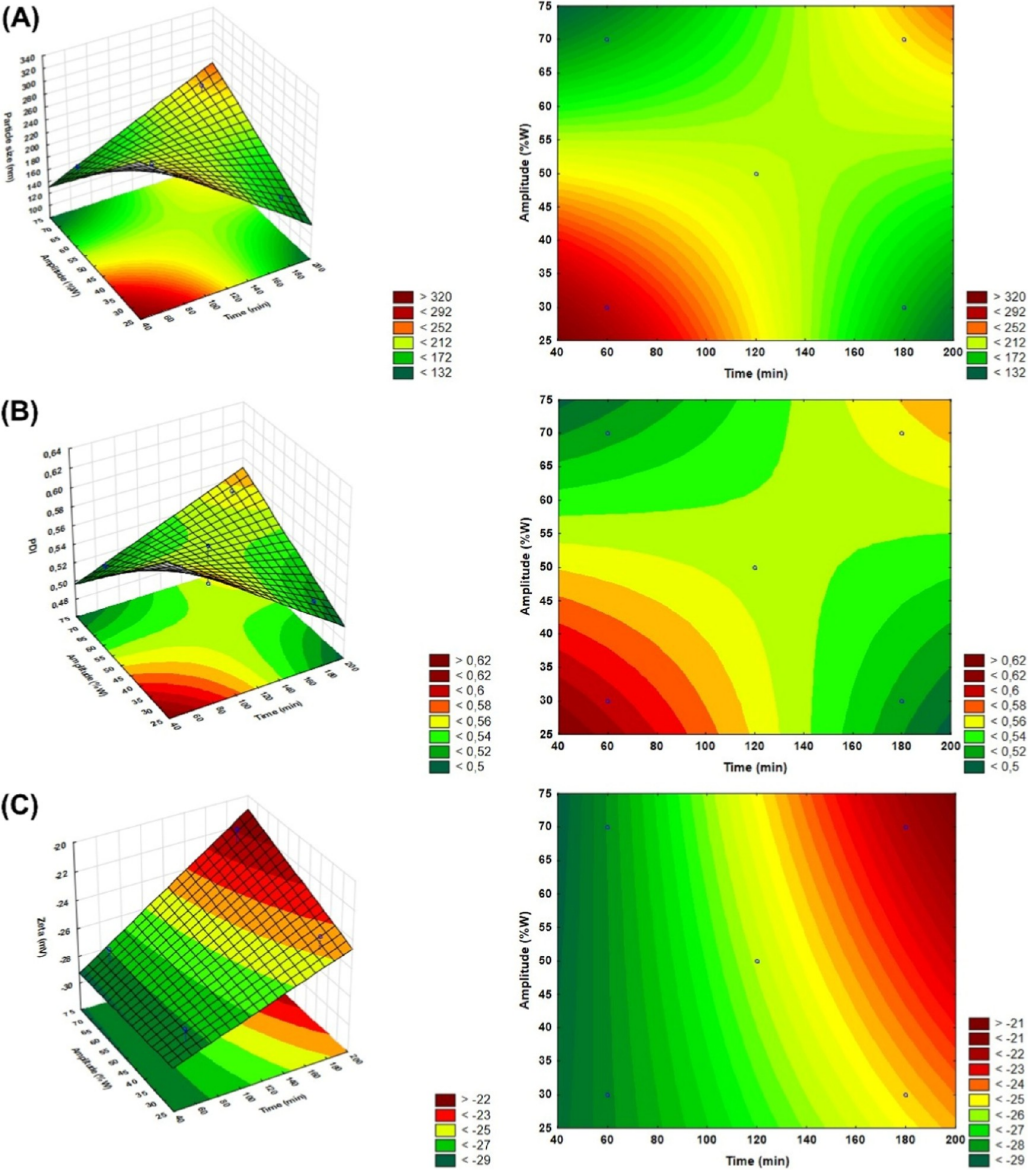
Response surfaces fitted with (A) particle size, (B) polydispersity index (PDI), and (C) zeta potential (ζ) as a function of ultrasonic cavitation parameters, time and amplitude.

Figure 4 shows the response surfaces illustrating the influence of the ultrasonication parameters (time and amplitude) on the particle size, polydispersity index (PDI), and zeta potential (ζ). Analysis of these results, corroborated by the Pareto diagrams (Figure 4), indicates that the combined effects of time and amplitude significantly influenced (*P* < 0.05) both the particle size employed (Figures 3A and 4A) and the PDI (Figures 3B and 4B). However, in the zeta potential (ζ), there were no significant variations in intensity by the individual or combined parameters (*P* > 0.05), as shown in Figures 3C and 4C. However, Figure 3C shows a predisposition of the time parameter to influence the intensity of ζ. The effects of the sonication parameters on the formation of hydrated carbon oxide nanostructures were subjected to a second-order multiple regression analysis. The assumptions of the model were checked using the Shapiro–Wilk and Kolmogorov–Smirnov tests. The significance of the regression model was assessed using one- way ANOVA. The resulting model equation for particle size (*Y*_PS_) is shown in eq 4.

4 $$Y_{PS}=594,47-2,76\left(time\right)-8,70\left(amplitude\right)+0,0026\left({time}^{2}\right)+0,0358\left({amplitude}^{2}\right)+0,0387\left(time\times amplitude\right)$$

The accuracy of the mathematical model can be seen from the significance of the regression of the variable *R* ^2^ = 0.8268. In this case, it is possible to obtain the response surfaces for the particle size in HSACO (Figure 4). Equation 4 shows that the variables (time and amplitude) negatively influence the response linearly. However, the quadratic and interaction effects show nonlinear and combined (and/or conditional) behavior. Although eq 4 shows that the individual effects are significant, the existence of an interaction element suggests that the response depends on the interaction of these factors. This makes it difficult to analyze the parameters in isolation. Therefore, the evaluation must consider the combined effects to understand the system’s behavior. This behavior corroborates the results presented in the Pareto diagram (Figure 3).

The time/amplitude interaction during ultrasonic cavitation has been shown to be effective in obtaining nanostructured materials with diameters predominantly less than 200 nm (Table 2 and Figure 4). However, it is important to consider that the application of high power and long sonication times can induce sintering processes and reagglomeration of the material.^44^ Martins Strieder et al.^30^ indicated that the mechanism of action caused by acoustic cavitation proved to be capable of reducing the particle size of suspensions and/or emulsions to obtain nanostructures. As a result, the particle size distribution in HSACO after the sonication process ensures better polydispersity of the material, guaranteeing the permanence of the nanometric structures, as shown in Figure 5.

**Figure 5:**
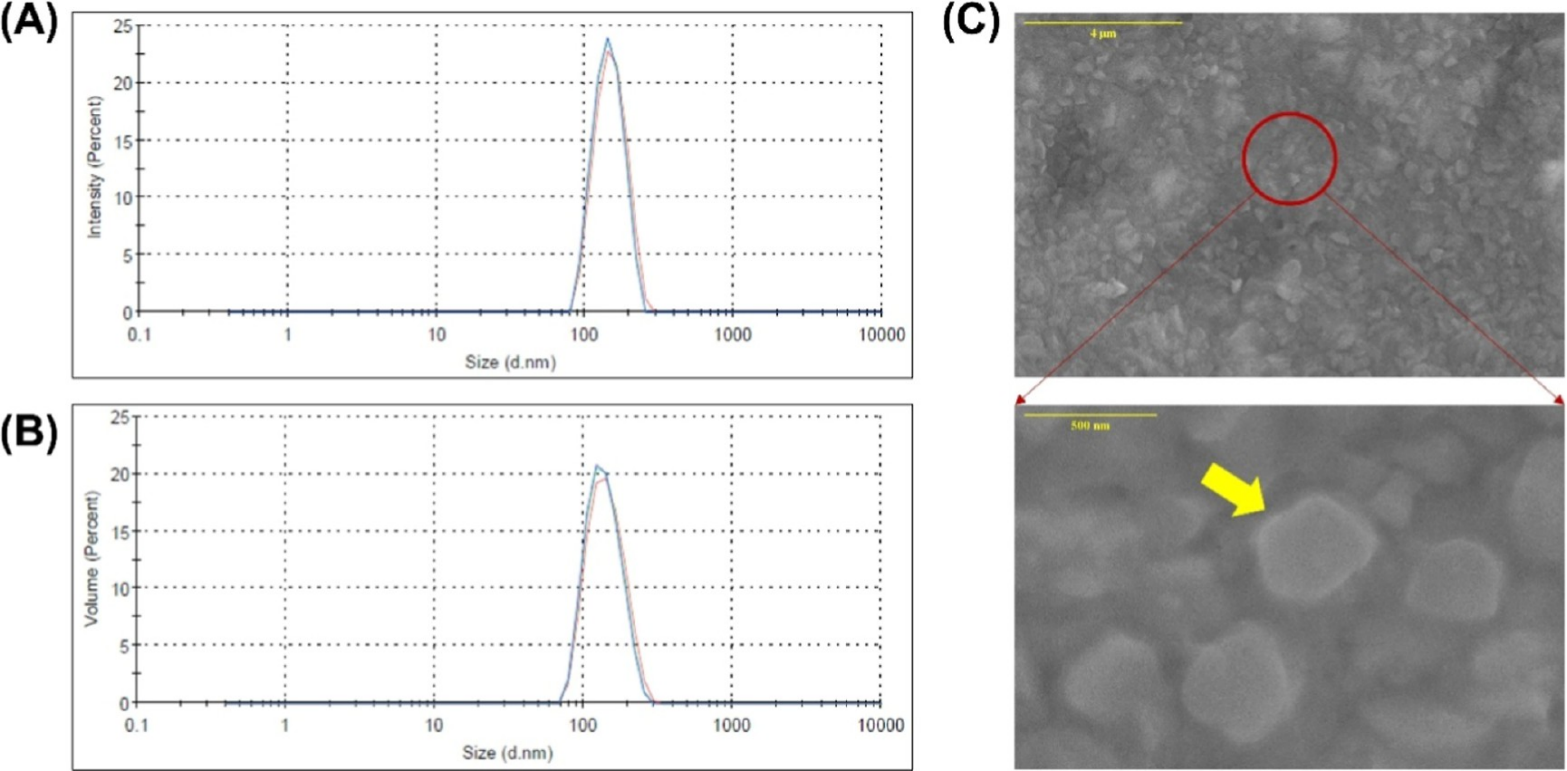
Particle size distribution by DLS in (A) intensity and (B) volume of HSACO-8. In (C), the micrograph of HSACO-8.

The particle size distributions obtained by DLS, shown in Figure 5A and B, exhibited an apparent monomodality profile, suggesting a main population of particles (close to 200–400 nm). However, when the two distributions are compared, the intensity distribution (Figure 5A) is more sensitive to the presence of larger particles due to their greater light scattering capacity, indicating the existence of a minority fraction of particles with sizes smaller than 100 nm. However, it predominates below 500 nm, contributing significantly to the signal. In contrast, the volume distribution (Figure 5B) tends to underestimate the contribution of these larger particles. This behavior, which suggests the coexistence of nanostructures with some larger aggregates, is visually corroborated by the micrograph shown in Figure 5C, which shows the existence of aggregated and overlapping materials.

The data observed in the sonic cavitation process corroborates the effectiveness of the method in obtaining nanostructures, as shown in Figures 4 and 5C. Previous research reported that DLS analysis is insufficient due to its limitations in obtaining conclusive results, as it is a comparative method with models of individual spherical particles with Brownian movement.^45^ Han Lyn et al.^45^ observed similar behavior when applying sonication to graphene oxide (GO) in their studies. These authors reported that GO nanosheets grouped were considered particles and that the carbon atoms with an sp^2^ structure may have been considered uncharged Lennard–Jones spheres. Thus, the behavior of the DLS analysis can be regarded as inconclusive in relation to the determination of particle sizes reported in the literature.^45^ To overcome the limitations of the analysis and validate the results obtained for carbon particle size, complementary techniques were employed including FEG-SEM (Figure 5C) and atomic force microscopy (AFM) (Figure 6). The integration of these analyses allowed for a comprehensive and reliable evaluation of the physical–morphological characteristics of the nanometric material. The choice of treatment 8 (30%W and 120 min) for this specific analysis was based on the results of the experimental design (Figure 4), which indicated that these sonication conditions were the most effective in obtaining particles with the smallest average size and a homogeneous PDI (PDI <0.3).

**Figure 6:**
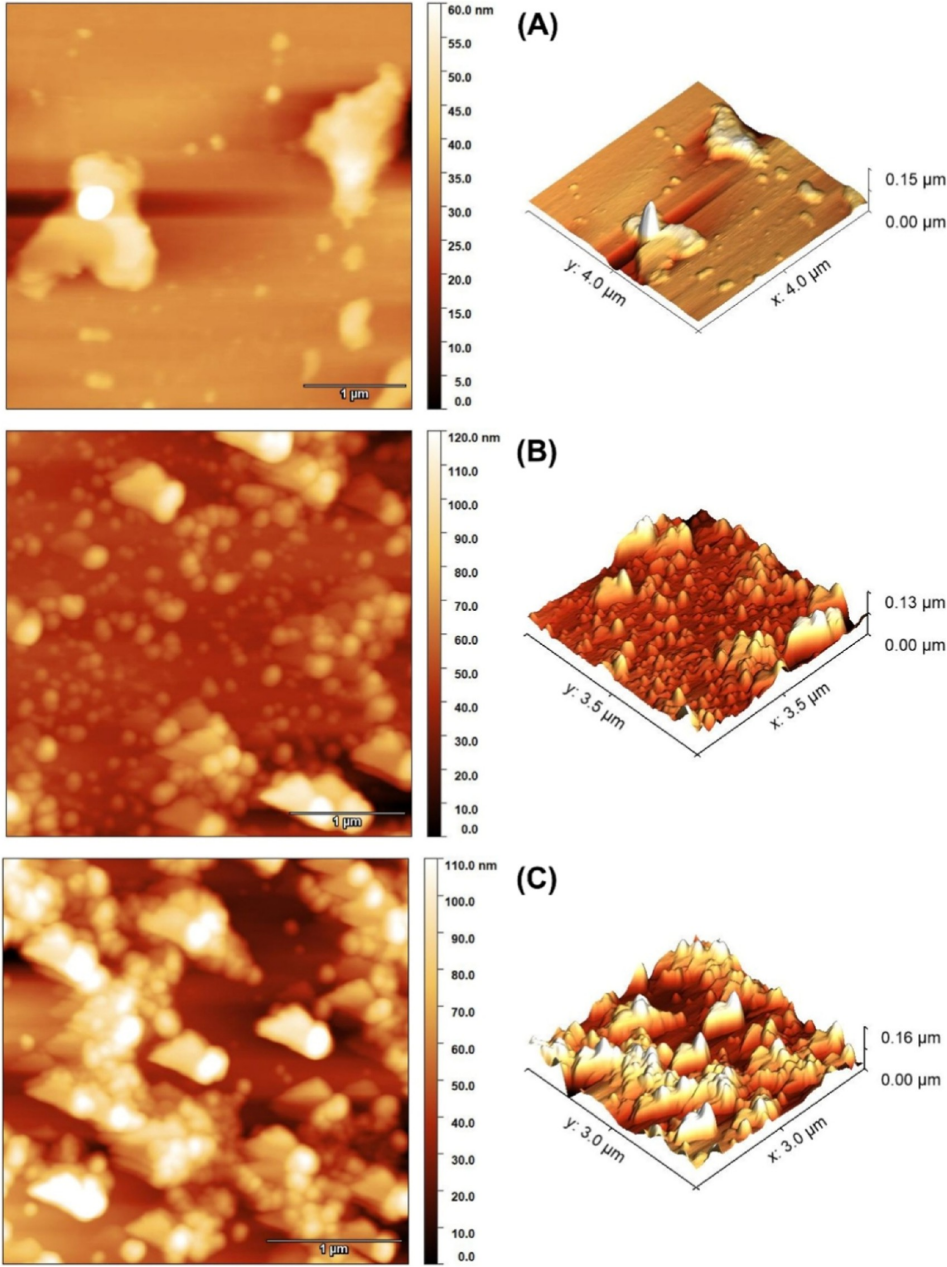
AFM micrographs of the 2D and 3D particle size distributions for (A) NHKBIO, (B) HSACO-8 treatment after sonication (30% W for 120 min), and (C) HSACO-8 after 24 h in standby of the sonic cavitation process in a tip ultrasound.

Figure 6 shows the presence of nanostructured materials and agglomerations of the material (Figure 6 in 3D), considering that HSACO has polydisperse behavior. During the sonic cavitation process, the hydrated amorphous carbon oxide (HACO) particulates fragmented, which may have been caused by the force of the microjets on its surface, causing a puncture effect.^45,46^ In addition, the literature indicates that prolonged sonication can promote material aggregation.^47,48^ This phenomenon is attributed to the increase in the concentration of fragments generated over the processing time, which leads to an increase in the frequency of interparticle collisions and, consequently, detachments, increasing the rate of reagglomeration.

Raman spectroscopy was used to assess the structural changes and degree of defects in the carbon material resulting from the sonic cavitation process. Recognized as a nondestructive technique, Raman spectroscopy is very powerful for characterizing carbonaceous materials, which is suitable for analyzing the defect levels and crystallinity based on the inelastic light scattering response.^49,50^ The Raman spectrum of the HSACO-8 sample, obtained after sonication and 24 h of rest, is shown in Figure 7.

**Figure 7:**
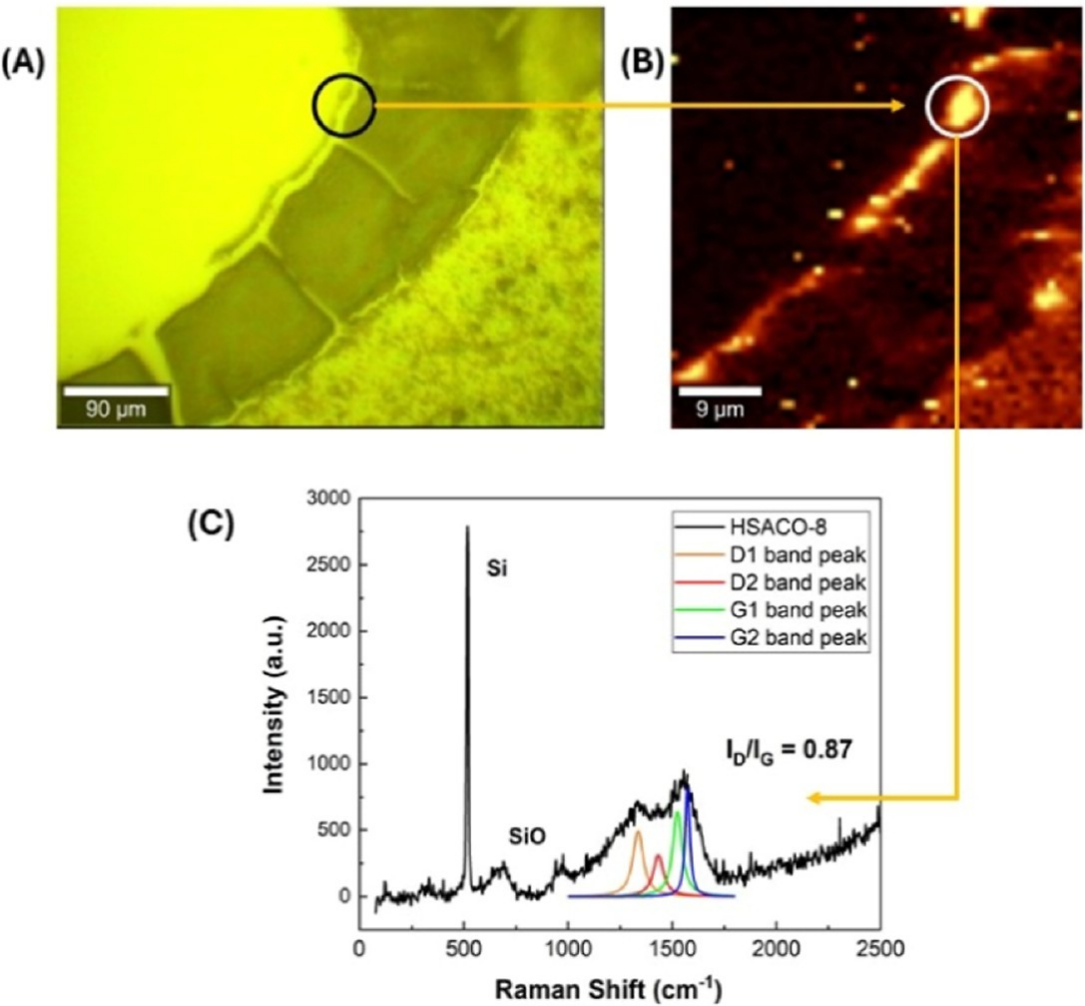
Optical micrograph of the region where the Raman mapping was performed (A), Raman intensity map of the G-band vibrational mode, in which brighter regions correspond to locations with higher G-band intensity (B), and Raman spectrum acquired from the highlighted area (circled region), together with the Lorentzian components used to fit the D and G bands (C) of the hydrated carbon oxide nanostructured material, HSACO-8 (after 24 h of rest). Raman spectra were acquired using a 532 nm Nd/YAG laser line.

The Raman spectrum, Figure 7C, displays a broad and asymmetric band spanning the 1200–1700 cm^–1^ region, which is characteristic of disordered and amorphous carbon materials.^51^ The G band, centered at approximately 1580 cm^–1^, exhibits significant broadening, reflecting a high degree of structural disorder within the sp^2^ carbon network and a wide distribution of bond lengths and bond angles.^52–55^ A pronounced D band is observed near 1350 cm^–1^, arising from defect-activated breathing modes of aromatic rings and indicating the presence of a substantial density of structural defects and small sp^2^ domains.^49,52,53^

To obtain a reliable quantitative analysis, the Raman spectrum was fitted using four Lorentzian functions in accordance with the Ferrari–Robertson model for amorphous carbon.^51^ Two Lorentzian components were assigned to the D band, accounting for different disordered sp^2^ configurations, while two additional components were used to describe the G band, including the D′ contribution associated with defect-induced intravalley scattering.^51^ The *I*_D_/*I*_G_ ratio was determined from the integrated areas of the fitted components, yielding a value of *I*_D_/*I*_G_ = 0.87, which is consistent with amorphous carbon in the stage II–III regime of the Ferrari–Robertson classification.^51^ In this regime, the *I*_D_/*I*_G_ ratio is governed by the degree of disorder and the size of sp^2^ clusters rather than by the crystallite size relationship described by the Tuinstra–Koenig model.^53^

In addition to the characteristic D and G bands, weak Raman features are detected at approximately 680 and 960 cm^–1^. These bands are not intrinsic to the sp^2^ carbon network and are attributed to vibrational modes associated with oxide-related species or oxygen-containing functional groups. Such contributions may originate from either surface oxidation of the carbon film or the underlying substrate, particularly in the case of oxidized silicon or glass substrates. Similar low-frequency Raman features have been reported in amorphous carbon films deposited on oxidized substrates and are commonly associated with M–O and Si–O vibrational modes, indicating the presence of extrinsic contributions to the measured spectrum.^56–58^ According to Santa Anna Santos et al.,^26^ the presence of Raman spectral bands between 600 and 1000 cm^–1^ can be attributed to the highly functionalized and hybrid nature of NHKBIO, where additional controls such as substrate Raman, FTIR, XPS, and isotopic exchange could be employed in future research as a way to consolidate the bands.

Electrochemical testing of carbon materials, such as graphite, graphene, and their oxides, can be used to provide information about the kinetic and thermodynamic properties of these materials. This can be achieved through the controlled injection or withdrawal of electrons using cyclic voltammetry.^55,59^ Figure 8 shows the cyclic voltammograms of (K_3_[Fe­(CN)_6_]) in an electrolytic medium with a characteristic duck-shaped profile.

**Figure 8:**
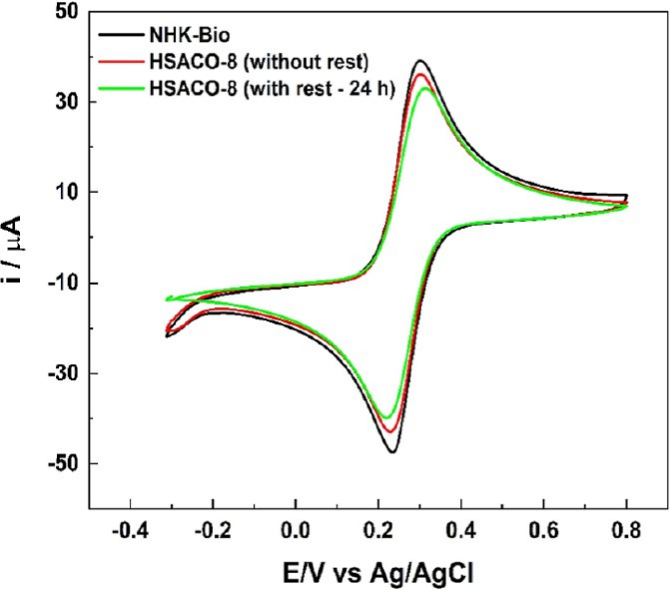
Cyclic voltammograms of NHKBIO (commercial product), HSACO-8 (after sonic cavitation, without resting), and HSACO-8 (after resting for 24 h) in a solution of K_4_Fe­(CN)_6_ (4 mmol/L) and KCl (1.0 mol/L) in the potential window from −0.3 to 0.8 V at 50 mV/s.

Figure 8 illustrates the conductive profiles of the materials above 30 μA, with 39.14 μA, 36.09 μA, and 33.03 μA observed for NHKBIO, HSACO-8 (without rest), and HSACO-8 (after 24 h of rest), respectively, indicating favorable conductivity and electrochemical responses. The cyclic voltammogram of the HSACO-8 sample after 24 h of rest (Figure 8) demonstrated a shift of the oxidation peak to a more positive potential (315 mV), accompanied by a reduction in the peak current. This behavior suggests an increase in the energy barrier of the redox process, with a possible association with the morphological changes observed in the material.^55^ The data presented in Figures 5 and 6, obtained using DLS and AFM, respectively, lend strong support to the hypothesis of particle nonuniformity and aggregation. Consequently, the electrochemical changes observed due to sonic cavitation after a 24 h rest period likely promoted morphological changes, thereby reducing the deposited electroactive area and the charge transfer efficiency of the samples during the redox process. In contrast, Ghanem and Rehim^55^ reported improved electrical conductivity in carbonaceous materials. The authors observed greater electron mobility in monolayer and multilayer graphene structures (lamellar morphology), demonstrating that structural differences and surface organization can influence the electrochemical behavior of the material. As illustrated in Table 3, the anodic (*I*_pa_) and cathodic (*I*_pc_) peak currents, along with the potential difference (Δ*E*_p_), are presented.

**Table 3:** *I*_pa_, *I*_pc_, and Δ*E*_p_ Values of the Samples in the Presence of (K_3_[Fe­(CN)_6_]) (4 mmol/L) and KCl (1.0 mol/L) Solutions

sample	*I*_pa_ (μA)	*I*_pc_ (μA)	*j*_pa_ (μA/cm^2^)^a^	*j*_pc_ (μA/cm^2^)^a^	Δ*E*_p_ (mV)
NHKBIO	39.14	–47.50	553.7	–671.9	68.36
HSACO-8 (without rest)	36.09	–42.93	510.6	–607.3	70.80
HSACO-8 (after 24 h)	33.03	–39.89	467.3	–564.3	95.22

a Normalization by electrode area: GCE of Ø 3.0 mm has area ≈0.07069 cm^2^.

As demonstrated in Table 3, the conductivity of NHKBIO > HSACO-8 (without rest) > HSACO-8 (after 24 h) is shown. Consequently, the observed increase in peak-to-peak separation (Δ*E*_p_) for HSACO-8 (after 24 h), from 70.8 mV to 95.22 mV, contradicts the anodic and cathodic peak currents, suggesting a slowdown in heterogeneous electron transfer kinetics or a reduction in the electroactive area.^60,61^ According to the extant literature, the presence of Δ*E*_p_ values greater than the theoretical 59 mV, when considering a reversible process, may be indicative of quasi-reversible or kinetically limited electron transfer. This behavior may result from a few factors, including (i) a reduction in the electroactive area; (ii) the formation of thicker films; or (iii) nonuniformity and particle aggregation resulting from the drop-casting technique.^62^ It is evident from the observations concerning the interfacial kinetics and inherent electrical conductivity exhibited by the materials (commercial NHKBIO and nanostructured HSACO-8, with and without rest) that the results were positive. This is primarily due to the substantial conductive capacity, as evidenced by peak currents above 30 μA (Table 3). Consequently, the observed changes are more indicative of alterations in morphology than of a loss of conductive properties.^63^

The combination of these properties (intrinsic electrical conductivity, morphological stability, and renewable origin) renders HSACO-8 a promising candidate for application in polymer matrices, forming conductive composites with potential applications in emerging packaging (smart and active).^64^ In this instance, the incorporation of conductive carbon fillers can result in the formation of percolating networks, thereby conferring an electrical response to films.^65^ In addition, there is the integration of emerging food preservation technologies, such as pulsed electric field processing (PEF).^66–68^ Furthermore, EVA (ethylene vinyl acetate) films, in conjunction with 30 wt % by weight of tobacco smoke particles, exhibited an electrical conductivity of 0.75 S m^–1^, accompanied by bacterial inactivation of 2.1 log 10.^67^ The same authors previously used PEF in-pack as a food packaging bag and obtained a microbial inactivation of 5.9 log 10, reaching the pasteurization level.^68^ Thus, functionalization of food packaging with bioactive materials that combine barrier characteristics, biodegradability, sustainability, and multifunctionality has the potential to result in innovations within food packaging sector.^65^

### 3.2 Acute Toxicity and Novel Tank Test

The biological safety assessment of HSACO and the pure material (NHKBIO) was performed using acute toxicity tests (96 h) and a novel tank test with adult zebrafish (*Danio rerio*). This in vivo model was selected due to its sensitivity and established validation for toxicological and neurobehavioral studies, enabling a robust analysis of the suspension’s effects.^33,68^

Acute toxicity results (Table 4) showed that HSACO dispersions at the tested concentrations (0.75%, 1.50%, 3.00% v/v), NHKBIO, and the controls did not induce significant mortality or anatomical changes in fish over 96 h (*P* > 0.05). Since no mortality was observed in any of the experimental or control groups (6 fish per group), no inferential statistical analysis was performed to compare the groups. The calculation of LC_50_ was not possible, since no mortality occurred even at the highest concentration tested (3.00% v/v), indicating that the LC_50_ is higher than this concentration.

**Table 4:** Acute Toxicity Tests on Adult Zebrafish up to 96 h with Different Concentrations of HSACO (v/v): 0.75% (D1), 1.50% (D2), and 3.00% (D3) (*n* = 6 Fish/Group)^a^

	HSACO (% v/v)			NHKBIO	H_2_O_Milli‑Q_*	H_2_O/C_2_H_5_OH**	96 h CL50 (%)/IV***
	D1	D2	D3				
mortality	0.00	0.00	0.00	0.00	0.00	0.00	–

a (*) Control: Milli-Q water; (**) 7.0% (v/v) hydroalcoholic solution; (***) LC_50_^—^lethal concentration to kill 50% of adult zebrafish; IV^—^confidence interval; probit (Log)*.

The behavioral data obtained in the new tank test were analyzed using GraphPad Prism v8.0 software. Initially, the data were assessed for normality using the Shapiro–Wilk test and for homogeneity of variances using Levene’s test. Once the assumptions were met, a one-way analysis of variance (ANOVA) was performed to compare the different experimental groups. When significant differences were identified, Tukey’s post hoc test was applied for multiple comparisons. The results were expressed as the mean ± standard deviation (SD), considering a significance level of *p* < 0.05. These findings indicate a low acute toxicity profile for the carbon material, a critical requirement for applications involving biological contact.

According to the literature, when zebrafish is introduced to a new environment, there is a likelihood of behavioral changes, such as anxiety, prolonged stay in selected parts of the tank, freezing behavior, and impaired locomotor actions.^33,69^ The novel tank test was used in this study to assess patterns of anxiety and locomotor activity in adult zebrafish treated with carbon at different concentrations (Figure 9).

**Figure 9:**
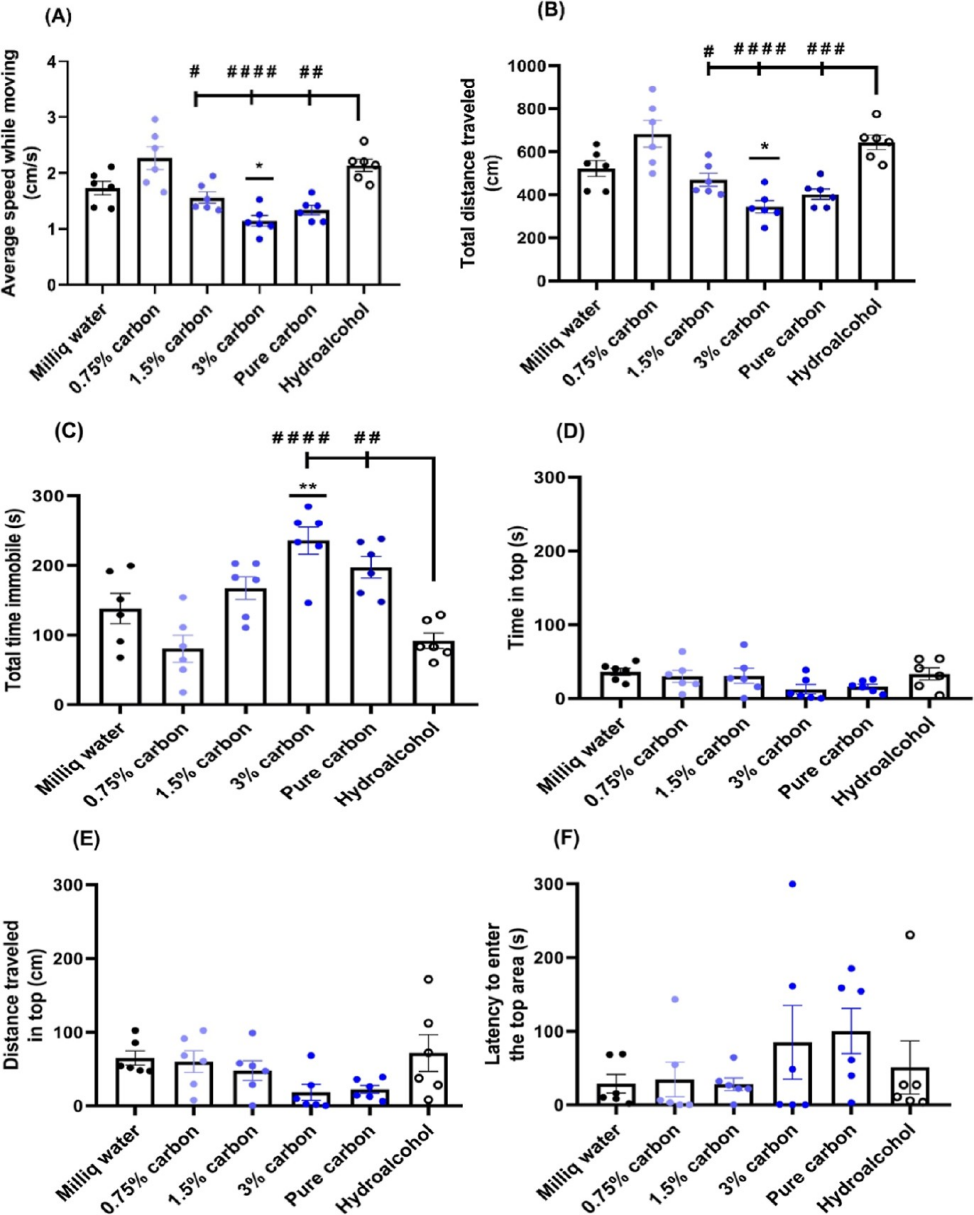
Effect of carbon on zebrafish locomotor activity and anxiety-like behavior in the Novel Tank test for 5 min (*n* = 6 fish/group). (A) Average speed in motion (cm s^–1^), (B) total distance traveled (cm), (C) total time immobile (s), (D) time at the top (s), distance traveled at the top (cm), and (E) latency to enter the top area (s). Data are expressed as the mean ± SEM. Each circle indicates individuals used in each treatment, and the asterisk above the bars indicates significance in comparison with the control group—Milli-Q water (*P ≤ 0.05; **P ≤ 0.01)—and the icicle indicates significance in comparison with the control group—hydroalcoholic solution (^#^ *P* ≤ 0.05; ^##^ *P* ≤ 0.01; ^###^ *P* ≤ 0.001; ^####^ *P* ≤ 0.0001).

A recent study has shown the importance of a more comprehensive toxicological approach to nanomaterials, incorporating cytotoxicity^70^ especially when these materials are obtained through sustainable routes. In this sense, studies investigating nanoparticles synthesized by green methods have demonstrated promising safety profiles. For example, metallic nanoparticles produced by green synthesis showed an absence of cytotoxicity in normal cells (VERO cells), did not induce oxidative stress, and even exhibited genoprotective effects, as evidenced by cell viability analysis, production of reactive oxygen species, nitric oxide, and extracellular DNA release. These results indicate that functionalized and properly stabilized nanomaterials can interact with biological systems without triggering significant cellular damage.

The novel tank test was used to evaluate anxiety and locomotor activity in adult zebrafish exposed to carbon at different concentrations (Figure 9). In this paradigm, behavioral phenotypes are quantified by measuring distance traveled, average speed, frequency of immobility episodes, and location/depth preferences.^36^

One-way ANOVA analysis revealed that the highest concentrations of carbon (NHKBIO and 3.00% v/v HSACO) caused motor impairment and increased immobility in adult zebrafish. HSACO at 3.00% reduced average speed and total distance traveled and increased immobility (*P < 0.05 and **P < 0.01, Figures 9A, 9B, and 9C, respectively) compared to the control group (Milli-Q water). Significant differences were also observed between the groups treated with the highest carbon concentrations and the hydroalcoholic solution group (^#^ *P* < 0.05; ^##^ *P* < 0.01; ^###^ *P* < 0.001; ^####^ *P* < 0.0001, Figures 9A and 9B). NHKBIO and HSACO at 3.00% v/v significantly increased immobility time compared to the hydroalcoholic solution group (^##^ *P* < 0.01; ^#####^ *P* < 0.0001, Figure 9C). However, the carbon concentrations did not alter the time spent at the top, distance traveled at the top, or latency (*P* > 0.05, Figure 9D–F).

In zebrafish, increased immobility and reduced locomotor activity are indicative of anxiety-like responses to a novel environment.^69,71^ Anxiolytic compounds typically increase the time spent in the upper region of the aquarium.^72^ In this study, the highest concentrations of HSACO (3.00% v/v) and NHKBIO induced inactivity and motor impairment, suggesting increased anxiety. In contrast, lower carbon concentrations did not elicit these behaviors.

Several studies have shown that exposure to nanomaterials can alter behavioral parameters in zebrafish, especially those related to locomotion and anxiety-like responses, indicating that sublethal effects on the nervous system can occur even in the absence of acute mortality. For example, silica nanoparticles (SiO_2_ NPs) induced an anxiety- and depression-like phenotype in adult zebrafish in the novel tank test, characterized by reduced total locomotion, decreased exploration of the upper zones of the tank, and increased freezing behaviors, suggesting anxiogenic effects of the tested material.^73^ Furthermore, exposure to polystyrene nanoplastics (PS-NPs) at ambient concentrations also led to changes in swimming patterns and a preference for remaining at the bottom of the tank, with elevated latency times to explore elevated areas, indicating increased behavioral anxiety, as well as locomotor alterations and instability in movement trajectories compared to control fish.^74^

Although no mortality or anatomical alterations were observed within 96 h, the highest carbon concentration consistently modified behavior, indicating motor impairment and heightened anxiety-like responses. These results suggest a low acute toxicity profile, but further investigation is needed to evaluate potential sublethal and long-term effects, particularly under prolonged exposure conditions, as sublethal behavioral changes warrant caution.

Although the HSACO evaluated in this study has a composition different from those of the metallic and metal oxide nanoparticles described in the literature, these findings provide relevant conceptual support for the interpretation of the results obtained. The absence of mortality and anatomical alterations in adult zebrafish, coupled with the behavioral changes observed only at the highest concentrations, suggests that HSACO has a low acute toxicity profile, while the effects detected are mainly related to sublethal behavioral changes and motor impairment and not too severe systemic toxicity. Even so, considering evidence from the literature on the cytotoxicity and ecotoxicity of green nanomaterials, it is essential to conduct further studies that evaluate the sublethal and long-term effects of HSACO, including complementary ecotoxicological and cytotoxic assays, for a more comprehensive environmental and biological risk assessment.

## 4 Conclusion

The ultrasonic cavitation process used to produce hydrated amorphous carbon oxide (HSACO) from NHKBIO efficiently generates nanostructured materials with amorphous, conductive, and stable properties, as demonstrated by a polydispersity index (PDI) of ≤0.30 and structural similarity to graphene oxide and reduced graphene oxide. The optimized conditions (HSACO-8) exhibited a stable dispersion of nanostructures, highlighting the potential of ultrasonic technology in the sustainable synthesis of carbon nanomaterials. Acute toxicity tests in *Danio rerio* did not reveal mortality or anatomical alterations at the tested concentrations, although higher doses induced behavioral changes, indicating a low acute toxicity profile but the need for further study on sublethal effects. NHKBIO and HSACO-8 exhibited electrical conductivity, which corroborates their use as conductive and reinforcing agents for food packaging. These results position these materials as promising candidates for extending the shelf life of sensitive foods, particularly in active and smart packaging applications. Future research should seek to refine process optimization and establish comprehensive safety frameworks for industrial deployment while further exploring how the integration of these nanocomposites influences the performance of diverse packaging matrices.

## Supplementary Material



## Notes

Distribution of the statistical factorial design for the coded variables amplitude (%W) and time (min) and the results of the acoustic power, specific energy, ultrasonic intensity, and particle size values applied in the experimental design of the HSACO sonic cavitation process (PDF)
