# Supplementary material for: Ultrasound-Assisted Processing of Amorphous Hydrated Carbon Oxide from Plant Origin: Properties and Biocompatibility of Conductive Nanocomposites as a Proposal for Food Packaging
Source: ACS Omega. 2026 Mar 17;11(12):19482–96. doi: 10.1021/acsomega.5c13160 (PMC13044692; doi:10.1021/acsomega.5c13160)
Supplement: Supplementary file 1 [file ao5c13160_si_001.pdf]

Ultrasound-assisted processing of amorphous hydrated carbon oxide from plant origin: properties and biocompatibility of conductive nanocomposites as a proposal for food packaging

Kelvi W. E. Miranda[1], Francislei S. A. Santos[2], Francisco Carlos C. S. Salomão[3], Gabrielle A. Freire[3], Maria Kueirislene A. Ferreira[3], Jane E. S. A. Menezes[3], Antonio G. Souza Filho[4], Melvin Pascall[5], Maria do Socorro R. Bastos[6], Lucicléia B. Vasconcelos[1*]*

[1]Laboratory of Research and Innovation in Plant Products and Packaging, Department of Food Engineering, Federal University of Ceara, 60356-000, Brazil.

[2]Department of Industrial Processes and Chemical Engineering, Federal Institute of Bahia, campus Salvador, 40110-150 Bahia, Brazil.

[3]Science and Technology Center, State University of Ceará, 62930-000 Fortaleza, CE, Brazil.

[4]Department of Physics, Federal University of Ceará, 60455-900 Fortaleza, CE, Brazil.

[5]Department of Food Science and Technology, The Ohio State University, 43210 Ohio, USA.

[6]Packaging Laboratory, Embrapa Tropical Agroindustry, Fortaleza 60511-110, Brazil.

* Corresponding authors: *Laboratory of Research and Innovation in Plant Products and Packaging, Department of Food Engineering, Federal University of Ceara, 60356-000, Brazil.* kelviwilmiranda@gmail.com (Kelvi W.E. Miranda) and lucicleia_barros@ufc.br (Lucicléia B. Vasconcelos)

Table S1. Distribution of the factorial statistical design for the coded variables: amplitude (%W) and time (min).

Treatment	Coded variables		Real variables	
	X_1	X_2	Amplitude (%W)	Time (min)
1	-1	-1	30	60
2	1	1	70	180
3*	0	0	50	120
4	-1	1	30	180
5	1	-1	70	60
6*	0	0	50	120
7	0	1	50	180
8	-1	0	30	120
9	1	0	70	120
10	0	-1	50	60
11*	0	0	50	120

(*) Corresponds to the central component of the experimental design.

Table S2. Values of acoustic power, specific energy, ultrasonic intensity, and particle size applied in the experimental design to the HSACO sonic cavitation process.

Amplitude (%W)	Time (min)	AP[1] (W)	SE[2] (kJ/g)	UI[3] (W/cm^2)
30	60	0.025 ± 0.007	1.05 ± 0.30	0.006 ± 0.001
70	180	0.062 ± 0.002	8.58 ± 0.30	0.016 ± 0.000
50	120	0.047 ± 0.009	4.39 ± 0.89	0.012 ± 0.002
30	180	0.013 ± 0.006	1.88 ± 0.89	0.004 ± 0.002
70	60	0.144 ± 0.013	6.69 ± 0.59	0.038 ± 0.003
50	120	0.047 ± 0.009	4.39 ± 0.89	0.012 ± 0.002
50	180	0.033 ± 0.004	4.60 ± 0.59	0.009 ± 0.001
30	120	0.013 ± 0.006	1.25 ± 0.59	0.004 ± 0.002
70	120	0.079 ± 0.003	7.32 ± 0.29	0.021 ± 0.000
50	60	0.113 ± 0.045	5.23 ± 2.07	0.030 ± 0.012
50	120	0.047 ± 0.009	4.39 ± 0.88	0.012 ± 0.002

([1]) Acoustic power; ([2]) Specific energy; ([3]) Ultrasound intensity, where mean values ± standard deviation (n = 2).
